# Assessment of virus and *Leptospira* carriage in bats in France

**DOI:** 10.1371/journal.pone.0292840

**Published:** 2023-10-20

**Authors:** Youssef Arnaout, Evelyne Picard-Meyer, Emmanuelle Robardet, Julien Cappelle, Florence Cliquet, Frédéric Touzalin, Giacomo Jimenez, Zouheira Djelouadji

**Affiliations:** 1 Lyssavirus Unit, Nancy Laboratory for Rabies and Wildlife, ANSES, Malzéville, France; 2 USC 1233-INRAE Rongeurs Sauvages, Risque Sanitaire et Gestion des Populations, VetAgro Sup, Marcy l’Etoile, France; 3 UMR ASTRE, CIRAD, INRAE, Université de Montpellier, Montpellier, France; 4 UMR EPIA, INRAE, VetAgro Sup, Theix, France; 5 School of Biology and Environmental Science, Science Centre West, University College Dublin, Dublin, Ireland; 6 CPEPESC Lorraine, Centre Ariane, Neuves-Maisons, France; University of Reunion Island, RÉUNION

## Abstract

With over 1,400 species worldwide, bats represent the second largest order of mammals after rodents, and are known to host major zoonotic pathogens. Here, we estimate the presence of pathogens in autochthonous bat populations. First, we set out to check our samples for PCR amplification efficiency by assessing the occurrence of inhibited PCR reactions from different types of bat samples with amplifying the housekeeping gene β-actin. Second, we investigated the presence of five targeted pathogens in a French bat population using PCR. We targeted viral RNA of *Canine distemper virus*, *Alphacoronavirus*, *Lyssavirus*, *Rotavirus* and bacterial *Leptospira* DNA. To do so, we screened for these viruses in bat faecal samples as well as in oropharyngeal swab samples. The presence of *Leptospira* was assessed in urine, kidney, lung and faecal samples. Results showed a frequency of inhibited reactions ranging from 5 to 60% of samples, varying according to the sample itself and also suspected to vary according to sampling method and the storage buffer solution used, demonstrating the importance of the sampling and storage on the probability of obtaining negative PCR results. For pathogen assessment, rotavirus and alphacoronavirus RNA were detected in *Myotis myotis*, *Myotis daubentonii*, *Myotis emarginatus* and *Rhinolophus ferrumequinum* bats. Rotaviruses were also detected in *Barbastella barbastellus*. The presence of alphacoronavirus also varied seasonally, with higher frequencies in late summer and October, suggesting that juveniles potentially play an important role in the dynamics of these viruses. *Leptospira* DNA was detected in *M*. *myotis* and *M*. *daubentonii* colonies. The 16S rRNA sequences obtained from *Leptospira* positive samples showed 100% genetic identity with *L*. *borgpetersenii*. Neither canine distemper virus nor lyssavirus RNA were detected in any of the tested samples. This study is the first to show the presence of *Leptospira* in autochthonous French bats in addition to coronavirus and rotavirus RNA previously reported in European autochthonous bats.

## 1. Introduction

The order Chiroptera (bats) is the second largest order of mammals after rodents. With over 1,400 chiropteran species identified to date, bats account for one fifth of all mammalian species worldwide. There are two suborders: Yangochiroptera, including 12 microbat families, and Yinpterochiroptera, including Pteropodidae and five microbat families (including Rhinolophidae) [1, 2]. Of a total of 51 bat species protected in Europe, France hosts 36 species from four different families: Rhinolophidae, Vespertilionidae, Molossidae and Miniopteridae [3, 4]. Bats have been the subject of diverse studies, both at the ecological level due to their essential role in ecosystem functioning [5] and regarding their role in animal and public (human) health issues [6–8]. Bats do indeed host a wide variety of microorganisms of bacterial, parasitic, fungal or viral origin [9–14]. Several studies suggest that bats play a role in the emergence and transmission of these pathogens [15–18]. For example, bats have been identified as a natural reservoir hosts of emerging viruses including Marburg virus, Hendra virus, Sosuga virus, Nipah virus and lyssaviruses [19–22]. Furthermore, some studies suggest that MERS-CoV, SARS-CoV-1 and SARS-CoV-2 originated in bats [23–25], and that other animal species, such as civets for SARS-CoV-1 and camels for MERS-CoV, are intermediate hosts for human infection [26–28]. Among viruses, thousands of bat-associated viral species belonging to at least 28 diverse viral families have been discovered or detected in 196 bat species [29]. Most of these viruses are host specific with limited zoonotic potential. For instance, astroviruses have been detected in bat populations, but no known case of transmission from bats to humans has been reported [28, 30]. Although the number of articles focusing on bats and viruses has been increasing with 3,918 studies retrieved from PubMed between 2000 and 2022, few studies have been undertaken on bat bacterial infections in France or Europe on the presence of *Bartonella* or *Leptospira* in bat populations [10, 31–33].

Today, the exact role of bats in the maintenance and transmission of some pathogenic microorganisms to hosts is still unclear. To investigate the presence of zoonotic pathogens in autochthonous bats in France, we conducted an exploratory study in close collaboration with bat workers. We targeted five different pathogen microorganisms, including four RNA viruses, canine distemper virus (CDV), alphacoronavirus (α-CoV), rotavirus, lyssaviruses and one bacterial disease agent, *Leptospira*.

The selection of these five pathogen microorganisms was based on a literature review of the studies carried out in neighbouring countries. Bats are commonly infected by α-CoV (β-CoV has also been identified in bat populations, but to a lesser degree) and lyssaviruses in Europe [6, 34–37] and rotavirus RNA has been episodically detected in metagenomics studies on bat faeces in Serbia in *Miniopterus schreibersii* [38] and in one dead bat in France, the bat being morphologically identified as *Myotis mystacinus* [9]. Although bats are known to harbour multiple paramyxoviruses, no studies have been undertaken on the presence of CDV in bats, well known for an ability to infect a broad range of host species and over time. Similarly, few studies reported the circulation of *Leptospira* in bats, although Leptospira was recently reported in dead bats in Czeck and Slovak Republics in Europe [39]. The epidemiological carriage of targeted pathogens and their circulation in bat species warrants deeper investigation.

During the last decade, PCR technology, i.e. conventional PCR as well as real-time PCR, has been widely used to detect and quantify pathogenic microorganisms that cause various infectious diseases. PCR, which is based on an enzymatic replication of nucleic acids, is sensitive to various mechanisms of inhibition [40] that can cause false negatives and high detection limits. Organic as well as inorganic substances can indeed be PCR inhibitors. Most of the known inhibitors are organic compounds, with for example bile salts, urea, lipids, complex polysaccharides, SDS as well different proteins such as collagen, haemoglobin or proteases [41, 42]. These inhibitors can be found in a variety of environmental samples (water, soil, etc.) as well as biological materials (including organs, blood, body fluids, etc.). Classical examples of PCR inhibitors include haemoglobin in blood, urea in urine and bile salts in faeces. Working on some types of biological matrix such as faecal samples or urine, rich in PCR inhibitors, is often complex. Many different strategies can be used to detect PCR inhibition to avoid false negative results, but there is currently no consensus for detecting PCR inhibition routinely. The first objective of this study was thus to verify that all collected samples are amplifiable before assessing the sample for the presence of pathogens. Therefore, we tested all samples with an endogenous control by amplifying the β-actin housekeeping gene in the sample.

Using molecular biology tools, we tested different types of biological samples, including kidney tissue, lung tissue, urine, faeces and oropharyngeal swabs collected in bats. Because CDV, α-CoV and rotavirus are transmitted by the faecal-oral route, and lyssavirus mainly by bites, we investigated the presence of viral RNA targets in faecal and oropharyngeal swab samples. *Leptospira* DNA was then screened for in urine, kidney, lung and faecal samples, because pathogenic leptospires colonize host kidneys and can be transmitted by contact with urine from infected animals or contact with contaminated water or environments [39].

The final goal of the present study was to provide a preliminary estimation of the presence of pathogens in bat populations in various matrices to explore the potential risks for humans and animals exposed to bats.

## 2. Materials and methods

### 2.1. Bat samples collection

In this study, we investigated the presence of pathogens in autochthonous bats using three different sampling protocols: 1) a longitudinal study of two bat colonies with the collection of faecal and urine samples; 2) a cross-sectional study with capture and release of bats during the swarming period and collection of faecal and oropharyngeal samples; 3) a collection of bat tissues (kidneys and lungs) on cadavers.

#### 1. Longitudinal study of two maternity colonies in eastern France (Grand Est, France)

**Fig 1:**
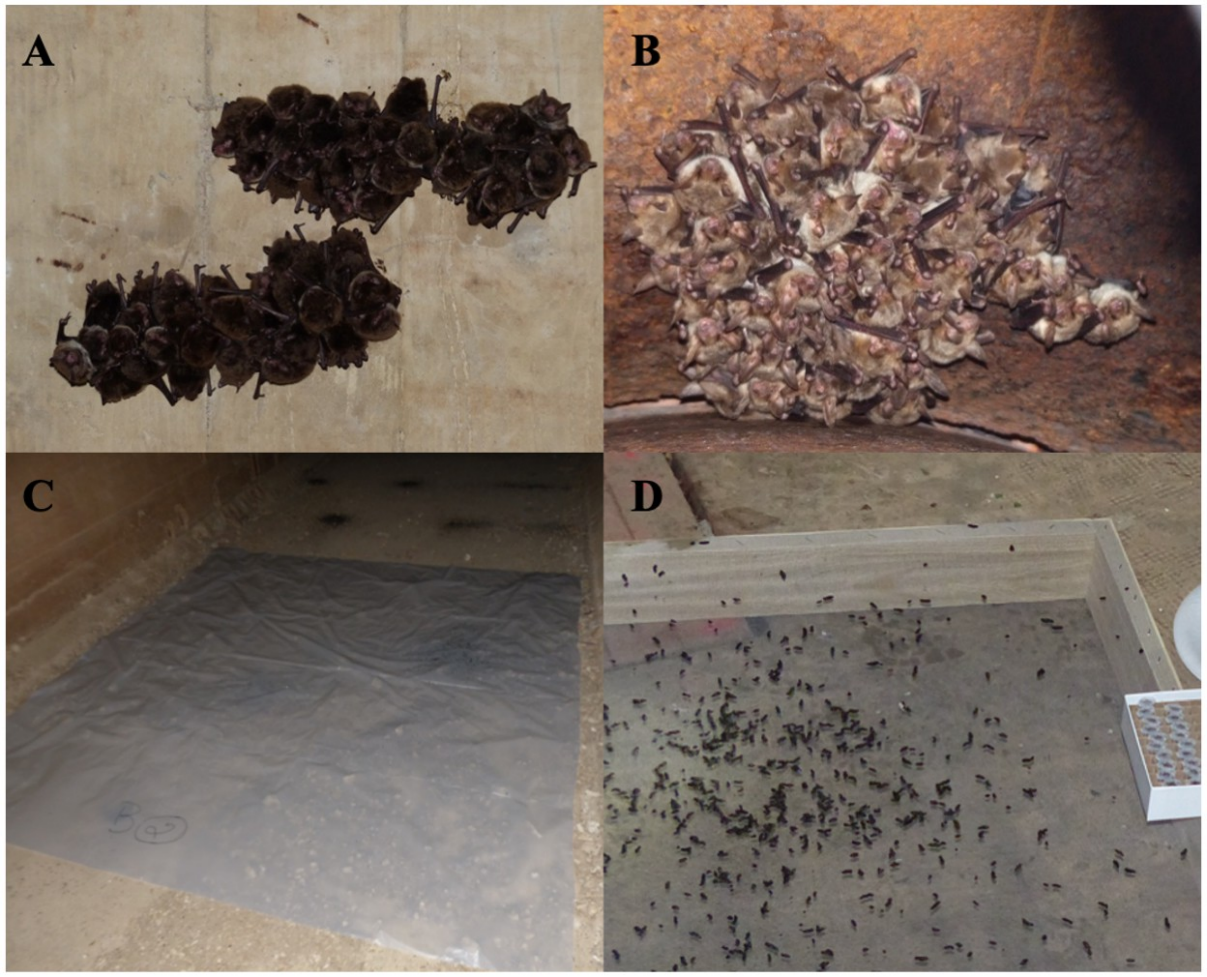
Picture of the maternity colonies of *Myotis daubentonii* at Site I (A) and *Myotis* at Site II (B). C: Depiction of the collection method used at Site I where faecal pellets were collected directly off the waterproof plastic tarp. D Depiction of the collection mode used at Site II where individual faecal pellets were collected off the mosquito net (used to prevent flooding of the sampling area).

The study was conducted at two different private sites, Site I (estacade) and Site II (fortification from the 1914s) for which all necessary authorizations were obtained. Each site hosted different autochthonous bat species, mostly *Myotis daubentonii* at Site I and mostly *Myotis myotis* at Site II. The two sites were separated by 39.7 km. Faecal and urine samples were collected during six consecutive months from May to October 2019, before and after parturition of bats. Sampling was carried out without any handling of bats by installing a waterproof plastic tarp (3 m^2^, 40 μm thick) on the ground under the colony on day 1 (D1) at each site. A homemade sampling device was deployed on the plastic tarp at Site II to prevent the flooding of the sampling area (Fig 1). This sample trap was 1 m^2^ mosquito net stretched over a wooden frame 10 cm above the ground. After the sampling session, the trap was reused after decontamination with the nucleic acid decontaminant DNA Away^™^ (Dutscher, Bernolsheim, France) and dried at room temperature until the next sampling session.

Fresh faecal and urine samples were collected on D0, ~24 h after installing the plastic tarp. The faecal samples were collected in DNase-/RNase-free tubes of 1.5 mL with one individual faecal pellet per tube; urine droplets present on the plastic film were pooled and collected using up and down with 1 mL of phosphate buffer saline (PBS),. All samples were stored at <-65°C until analysis.

#### 2. Transversal study through capture and release of bats during swarming (mating period) in western France (Brittany)

Bats were captured by authorized naturalists who had all the required authorizations for the capture and release of bats. Faecal and oral swabs were collected between September and October in 2017 and 2018. Bats were caught during the night with a harp trap, kept in individual bags until sampling and then released at the capture site. After each use, the bag was decontaminated and cleaned before re-use. Faecal samples were directly collected in bags and stored in DNase-/RNase free tubes. Oropharyngeal swabs were collected using sterile polyester swabs (Labellians, Nemours, France). Oropharyngeal swabs collected in 2017 were stored in DNase-/RNase-free tubes containing 500 μL of RNA Later (ThermoFisher Scientific, Dardilly, France) and oropharyngeal swab samples collected in 2018 were stored in DNase-/RNase-free tubes containing 500 μL of Dulbecco’s modified Eagle medium ((DMEM), ThermoFisher Scientific, Dardilly, France).

A total of 100 and 100 faecal samples were collected in 2017 and 2018, respectively in addition to the 194 and 36 oropharyngeal swabs collected in 2017 and 2018, respectively. All samples were stored at <-65°C until analysis. Trap location (municipality), year, identified species, gender (male/ female) and age (juvenile/adult) were recorded during the capture sessions.

#### 3. Collection of kidney and lung tissues from bat carcasses

A total of 157 bat carcasses, received as part of bat rabies passive surveillance at the Nancy Laboratory for Rabies and Wildlife, were necropsied between 2018 and 2019. All bats were previously diagnosed negative for rabies. A total of 109 lung and 157 kidney samples were collected in 1.5 mL DNase-/RNase-free tubes and stored at <-65°C until analysis. For 100 of the 157 bat carcasses, wing punches were used to genetically identify the samples based on amplification of the partial cytochrome b gene [43].

### 2.2. Ethics statement

All bat species are protected in Europe and in France. Therefore, all biological samples used in this study were collected for rabies diagnosis by the Nancy Laboratory for Rabies and Wildlife in compliance with the formal authorization granted by the French Ministry for the Environment. In France and within the European Union, the legal framework for the protection of animals used for scientific purposes is governed by Regulation 2010/63/EU of the European parliament and of the Council of 22 September 2010 (applicable and translated in French in 2013).

All captures and sample collections were carried out in accordance with the ethical guidelines and permits delivered in ‘Arrêté’ by the Préfet du Morbihan, Bretagne awarded to Frédéric Touzalin, for the time period 2017–2018. Access to the field sites was granted by local authorities in collaboration with Bretagne Vivante. All samples (saliva and guano) taken from live bats were taken in accordance with regulations and respect for animal welfare (Ethic Committee ANSES/ENVA/UPEC, n° 16).

### 2.3. Bat species identification

Morphological species identification was carried out by naturalists for each captured bat and for each bat carcass according to the illustrated identification keys of European bats described in Dietz et al. [44].

### 2.4. Detection of viral RNA and bacterial DNA

Before carrying out the viral and bacterial analyses, all bat samples included in the present study were simultaneously tested for the presence of the β-actin housekeeping gene. Two different β-actin amplifications were carried out. A β-actin RT-PCR was performed on RNA extracts before virological analysis (because we screened for RNA viruses) and a β-actin PCR was performed on DNA extracts before bacterial analysis. This amplification step, if successful, provided sufficient evidence of sample quality to detect pathogens using PCR and thus confirm the absence of inhibitors. The details on the PCR protocols for β-actin and each pathogen tested (α-CoV, rotavirus, lyssavirus, CDV and *Leptospira*) are described in Supplementary Materials, as well as the protocol for the nucleic acid extraction (RNA and DNA extraction) performed for each type of biological sample tested. The amplification of lyssavirus, rotavirus and CDV RNA was performed using real-time RT-PCR according to the protocols described in Supplementary Materials. CoV RNA was detected by amplifying a 438 bp fragment of the CoV RNA-dependent RNA polymerase (RdRP) gene using degenerate primers described in Gouilh et al. in 2011 [45]. Primers were previously designed based on a multiple alignment of the highly conserved nucleotide sequences of the coding fragment of the subunit of the RdRp: *nsp12* gene for the amplification of α-CoV and β-CoV (Pan-CoV RT-PCR) [45]. Real-time PCR was performed for *Leptospira* according to Merien et al. targeting the partial 16S RNA gene [46]. The protocol is described in Supplementary Materials. All primers and probes used in this study are described in S1 and S2 Tables (S1 File).

### 2.5. Real-time PCR and data interpretation

If β-actin housekeeping RNA was not detected, the sample was identified as not-amplifiable and was not considered for the viral pathogen analysis to avoid false negative results. Similarly, if β-actin housekeeping DNA was not detected, the sample was identified as not-amplifiable and was not considered for the *Leptospira* analysis.

Therefore, a sample was reported positive for the presence of a pathogen when this sample was tested positive for β-actin as well as for the presence of the targeted pathogen. Based on prior laboratory experiments, cut-off values of < 45 and < 33 Ct were used to define positive results of virological and *Leptospira* analysis, respectively.

### 2.6. Electrophoresis and sequencing

#### 2.6.1. RNA and DNA detection

For RNA virus detection, amplicons were analysed using 2% agarose gels stained with the intercalant SYBR Safe (ThermoFisher Scientific, Illkirch, France), then visualized using Bioimager (Bio-Rad, Roanne, France).

For *Leptospira* identification, amplicons were analysed using 1.5% agarose gels stained with GelRed^®^ nucleic acid stain (Sigma-Aldrich, Saint Quentin-Fallavier, France).

#### 2.6.2. Sequencing and phylogenetic analysis

PCR products were Sanger sequenced by a service provider (Eurofins, Ebersberg, Germany for RNA and Genoscreen, Lille, France for DNA) with the reverse and forward primers used in the PCRs. All nucleotide sequences were assembled using Vector NTI software (version 11.5.3) (Invitrogen, France) and ChromasPro (version 2.6.6). Sequence alignments and determination of the percentages of identities and similarities were carried out using BioEdit Software (version 7.2.5) and MEGA X (version 10.1.8). Preliminary genetic identification was determined using BLAST (Basic Local Alignment Search Tool). A phylogenetic tree was constructed only for coronaviruses using the PhyMl method (HKY 85 model) with SeaView by comparing 63 referenced sequences representing the four coronavirus genera and 28 sample sequences from this study (S3 Table in S1 File). The bootstrap probabilities of each node were calculated using 1000 replicates to assess the robustness of the maximum likelihood method.

### 2.7. Statistical analysis

First, proportions of non-amplifiable samples (according to RNA and DNA β-actin results) were computed. Amplifiable samples were used to estimate the prevalence of pathogens among the different sampling protocols and according to the different studied variables. For a given pathogen, non-interpretable results, when they occurred, were excluded from the analysis.

1. Longitudinal study of two maternity colonies: The proportions of non-amplifiable samples for both RNA and DNA analysis were calculated according to the type of "biological matrix" (faeces/urine). For faecal samples, the proportions of non-amplifiable samples were also assessed according to the "collection mode" (homemade trap deployed at Site II and not at Site I). For pathogen detection, the proportions of positive samples were calculated according to "biological matrix" (faeces/urine), "site" (with Site I and Site II corresponding to a specific bat species) and "month of collection" (May to October).
2. Transversal study through capture/release of bats during swarming: The proportions of non-amplifiable samples were calculated according to the type of "biological matrix" (faeces/oropharyngeal swabs). For oropharyngeal swab samples, the proportions of non-amplifiable samples were also calculated according to the "storage buffer" used (RNA Later used in 2017, DMEM used in 2018). For pathogen detection, the proportions of positive samples were compared according to the "biological matrix" (faeces/oropharyngeal swabs), and also according to environmental and biological factors "municipality", "year of capture" (2017 or 2018), "species captured", "age"(juvenile/adult) and "gender" (male/female).
3. Collection of kidney and lung tissue from bat carcasses: The proportions of non-amplifiable samples and positive samples for pathogens were compared between the different "biological matrices" (kidneys/lungs).

The comparisons of prevalence were carried out using Pearson’s chi-squared test (*χ*^2^) or the Fisher exact test when frequencies were below 5 [47]. All proportions were indicated with their 95% confidence intervals (95CI) calculated in R Studio software (version 1.4.1106) by using the exact binomial test.

## 3. Results

### 3.1. Detection of targeted viral RNA pathogens

A total of 696 faecal samples and 230 oropharyngeal swabs were analysed for the presence of the housekeeping RNA gene. β-actin RNA was not detected in 287 out of 696 faecal samples and in 123 out of 230 oropharyngeal samples, representing 41.2% (95CI [37.5–44.9]) and 53.4% (95CI [46.8–60.0]) of non-amplifiable samples, respectively. Amplifiable and not-amplifiable samples detected within the different sampling protocols are described in Table 1.A1.

**Table 1:** Detection of amplifiable/non-amplifiable samples. Table A1 gives β-actin RNA analysis performed before virological assessment, and Table A2 gives β-actin DNA analysis performed before *Leptospira* screening. A sample positive for β-actin means the sample is amplifiable and a negative β-actin sample means the sample is considered non-amplifiable.

**A1**									
						β-actin RNA detection			
Sampling protocol		Analysed matrix	Total	Sample collection method	Storage buffer	n amplifiable samples (%)	95% CI	n non-amplifiable samples (%)	95% CI
Maternity colonies	Site I (*M*. *daubentonii*)	Faeces	331	Plastic tarp	/	182 (54.9%)	[49.4–60.4]	149 (45.0%)	[39.5–50.5]
	Site II (*M*. *myotis*)	Faeces	165	Plastic tarp + mosquito net	/	129 (78.1%)	[71.0–84.2]	36 (21.8%)	[15.7–28.9]
Swarming period		Faeces	200	Individual bag	/	98 (49.0%)	[41.8–56.1]	102 (51.0%)	[43.8–58.1]
		Oropharyngeal swabs	194		RNA Later (2017)	76 (39.1%)	[32.2–46.4]	118 (60.8%)	[53.5–67.7]
			36		DMEM (2018)	31 (86.1%)	[70.5–95.3]	5 (13.8%)	[4.6–29.4]
**A2**									
						β-actin DNA detection			
Sampling protocol		Analysed matrix	Total	Sample collection method		n amplifiable samples (%)	95% CI	n non-amplifiable samples (%)	95% CI
Maternity colonies	Site I (*M*. *daubentonii*)	Faeces	329	Plastic tarp		273 (82.9%)	[78.4–86.8]	56 (17.0%)	[13.1–21.5]
		Urine	8			7 (87.5%)	[47.3–99.6]	1 (12.5%)	[0.3–52.6]
	Site II (*M*. *myotis*)	Faeces	161	Plastic tarp + mosquito net		153 (95.0%)	[90.4–97.8]	8 (4.9%)	[2.1–95.5]
		Urine	4			3 (75.0%)	[19.4–99.3]	1 (25.0%)	[0.6–80.5]
Bat cadavers		Lung tissue	109	/		58 (53.2%)	[43.4–62.8]	51 (46.7%)	[37.1–56.5]
		Kidney tissue	157	/		84 (53.5%)	[45.3–61.4]	73 (46.4%)	[38.5–54.6]

Abbreviations: 95% CI: 95% confidence interval; /: Not applicable; DMEM, Dulbecco’s modified Eagle medium

Of the four (α-CoV, rotavirus, CDV and lyssavirus) viral pathogens screened in faeces and oropharyngeal swabs, α-CoV and rotavirus were detected in faecal samples (27.8% 95CI [23.3–32.7] positive for α-CoV and 0.6% 95CI [0.0–2.0] positive for rotavirus) and in oropharyngeal swabs (1.0% 95CI [0.0–5.6] α-CoV positives and 15.2% 95CI [8.4–24.7] rotavirus positives). Neither CDV nor lyssavirus was detected in any sample, either in faeces or in oropharyngeal swabs. Positive samples detected with the different sampling protocols are described in Table 2.

**Table 2:** Results of pathogen screening in bats from various matrices collected within different protocols.

			α-coronavirus		Rotavirus		Lyssavirus		Canine distemper virus		*Leptospira*	
Sampling protocol		Analysed matrix	n positive /total* (%)	95% CI	n positive/total* (%)	95% CI	n positive/total* (%)	95% CI	n positive/total* (%)	95% CI	n positive/total* (%)	95% CI
Maternity colonies	Site I	Faeces	**57/164 (34.7%)**	**[27.5–42.5]**	**1/170 (0.5%)**	**[0.0–3.2]**	0/180 (0.0%)	[0.0–2.0]	0/180 (0.0%)	[0.0–2.0]	0/273 (0.0%)	[0.0–1.3]
		Urine	/	/	/	/	/	/	/	/	**2/7 (28.5%)**	**[3.6–70.9]**
	Site II	Faeces	**36/122 (29.5%)**	**[21.5–38.4]**	**1/120 (0.8%)**	**[0.0–4.5]**	0/129 (0.0%)	[0.0–2.8]	0/129 (0.0%)	[0.0–2.8]	0/153 (0.0%)	[0.0–2.3]
		Urine	/	/	/	/	/	/	/	/	**1/3 (33.3%)**	**[0.8–90.5]**
Swarming period		Faeces	**10/84 (11.9%)**	**[5.8–20.8]**	0/59 (0.0%)	[0.0–6.0]	0/37 (0.0%)	[0.0–9.4]	0/98 (0.0%)	[0.0–3.6]	/	/
		Oropharyngeal swabs	**1/96 (1.0%)**	**[0.0–5.6]**	**13/85 (15.2%)**	**[8.4–24.7]**	0/92 (0.0%)	[0.0–3.9]	0/107 (0.0%)	[0.0–3.3]	/	/
Bat cadavers		Lungs	/	/	/	/	/	/	/	/	0/58 (0.0%)	[0.0–6.1]
		Kidneys	/	/	/	/	/	/	/	/	0/84 (0.0%)	[0.0–4.2]

Abbreviations: 95% CI: 95% confidence interval; /: not tested;

* number of tested samples corresponds to amplifiable samples excluding non-interpretable samples

#### 3.1.1. Study of two maternity colonies

*3*.*1*.*1*.*1*. *Detection of* β-actin *RNA*. Of 496 faecal samples collected in both sites, 185 (37.3% 95CI [33.0–41.7]) samples tested negative for the presence of the *β-actin* RNA and consequently considered as non-amplifiable samples (Table 1.A1). No significant difference in non-amplifiable samples was detected between the different biological matrices (faecal and urine samples). However, the proportion of non-amplifiable faecal samples differed significantly between the two sites, which had different collection methods (homemade sampling trap and plastic tarp at Site II and only plastic tarp at Site I) (p*χ*^2^ = 4.82E-7), with a lower proportion of non-amplifiable faecal samples found at Site II (22.0% 95CI [15.7–28.9]) than at Site I (45.0% 95CI [42.2–53.6]).

*3*.*1*.*1*.*2*. *Detection of viral pathogens*. Out of 286 faecal samples collected at the two sites (samples tested positive for the *β-actin* RNA), 93 samples tested positive for α-CoV, corresponding to 32.5% (95CI [27.1–38.2]) of positive samples. Two faecal samples tested positive for rotavirus, corresponding to 0.6% (95CI [0.0–2.5]) positive samples (Table 2). Neither CDV nor lyssavirus was detected in any of the 286 exploitable samples, non-interpretable samples excluded.

**Fig 2:**
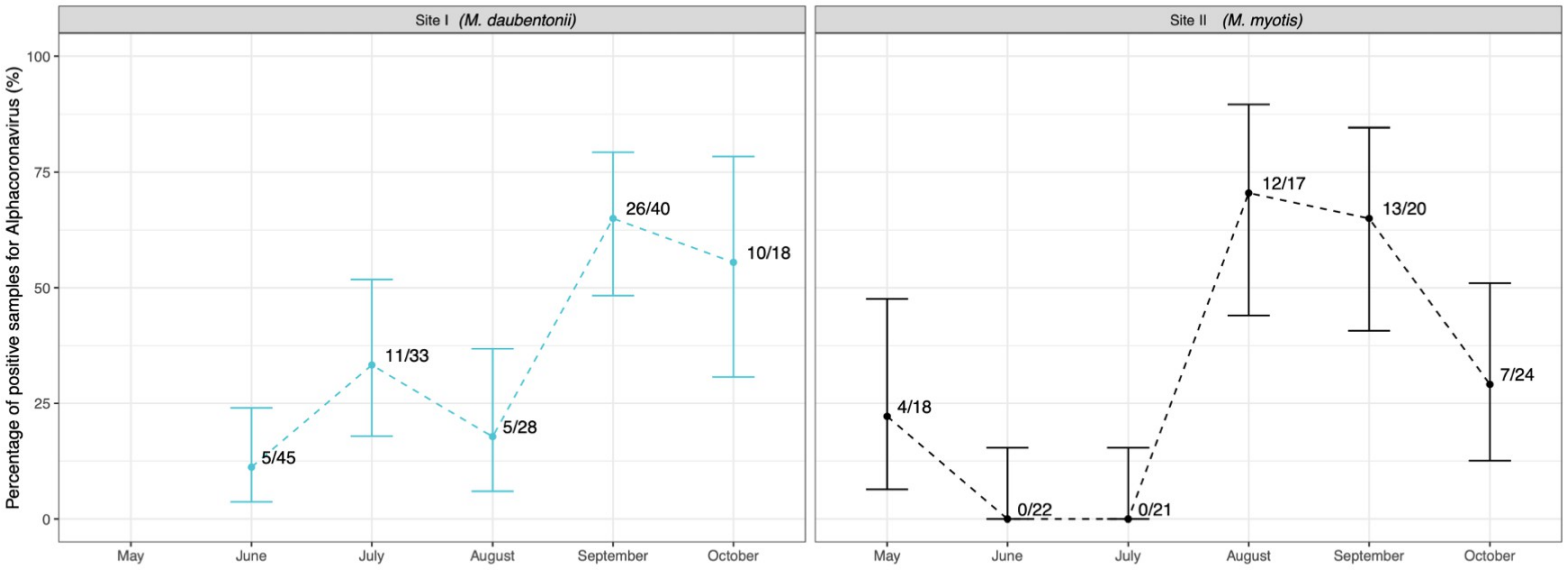
Temporal evolution of alphacoronavirus (α-CoV) detection in bat faecal samples collected in two maternity colonies (Site I and Site II).

At Site I hosting *M*. *daubentonii*, 57 faecal samples out of 164 tested positive for α-CoV (34.7% 95CI [27.5–42.5]). The monthly prevalence of α-CoV is shown in Fig 2. Interestingly, a peak was detected from September to October, with 26 (65.0% 95CI [48.3–79.3]) and 10 (55.5% 95CI [30.7–78.4]) faecal positive samples, respectively. The proportion of positive α-CoV samples differed significantly between the September–October period and the June-August period (p*χ*^2^ = 5.54E-8), with the highest prevalence being observed in September–October.

At Site II hosting *M*. *myotis*, 36 faecal samples out of 122 (amplifiable samples, non-interpretable excluded) tested positive for α-CoV (29.5% 95CI [21.5–38.4]). At this site, α-CoV prevalence also showed temporal variation. Significant differences were observed between the August–October period compared with the May–July period (p*χ*^2^ = 2.21E-8), with the highest prevalence being observed in August–October.

#### 3.1.2. Study of bats during swarming period

*3*.*1*.*2*.*1*. *Detection of β-*actin *RNA*. Out of 200 faecal and 230 oropharyngeal swab samples, 102 (51.0% 95CI [43.8–58.1]) faecal and 123 (53.4% [46.8–60.0]) oropharyngeal swab samples tested negative for the presence of β*-actin* RNA and considered as non-amplifiable samples (Table 1.A1). No significant differences were detected when comparing the proportions of non-amplifiable samples from the different biological matrices (faecal and oropharyngeal swab samples).

For oropharyngeal swab samples, 118 samples tested negative for β-actin RT-PCR out of 194 (60.8% 95CI [53.5–67.7]) collected in 2017 (stored in RNA Later buffer) whereas 5 samples tested negative for β-actin RT-PCR out of 36 collected in 2018 (13.8% 95CI [4.6–29.4]) (stored in DMEM). The highest proportion of non-amplifiable samples was observed in 2017. These proportions differed significantly according to the Pearson’s chi-squared test (p *χ*^2^ = 2.16E-7).

*3*.*1*.*2*.*2*. *Detection of viral pathogens*. For the study of α-CoV carriage, one sample (1.0% 95CI [0.0–5.6]) out of the 96 oropharyngeal swab samples (amplifiable samples, non-interpretable excluded) tested positive, whereas 10 out of the 84 amplifiable faecal samples tested positive (11.9% 95CI [5.8–20.8]) (Table 2).

For the study of rotavirus carriage, 13 out of the 85 oropharyngeal swab samples (amplifiable samples, non-interpretable samples excluded) tested positive (15.2% CI [8.4–24.7]). The 13 positive samples that were also tested using conventional RT-PCR with primers targeting the *nsp4* gene and described for the detection of rotavirus RNA in rabbits [48], tested negative and thus were not genetically typed. No rotavirus RNA was detected in amplifiable faecal samples. Neither CDV or lyssavirus were detected in faeces or in oropharyngeal swab samples (Table 2).

Interestingly, the proportion of positive α-CoV and rotavirus samples differed significantly according to the biological matrix (faecal or oropharyngeal swab samples) (p (Fisher) = 0.003 and 0.0008, respectively). Higher proportions of α-CoV-positive samples were found in faeces (11.9% 95CI [5.8–20.8]) than in oropharyngeal swabs (1.0% 95CI [0.0–5.6]), whereas a higher proportion of rotavirus-positive samples were found in oropharyngeal swab samples (15.2% CI [8.4–24.7]) compared with faeces samples (0.0% CI [0.0–6.0]).

The detection of α-CoV and rotavirus and their association with bat species is detailed in Table 3. α-CoV and rotavirus was found in four species: *M*. *myotis*, *M*. *daubentonii*, *Myotis emarginatus* and *Rhinolophus ferrumequinum*. Rotavirus was also detected in *Barbastella barbastellus* (Table 3). For the other bat species, differences could not be assessed for α-CoV and rotavirus due to the small sample sizes in each species. No significant variations in α-CoV and rotavirus presence were detected according to municipality, year, age or gender.

**Table 3:** Alphacoronavirus and rotavirus results obtained on the various bat species trapped during swarming. Positive results are shown in bold.

Bat species	Alphacoronavirus detection in bat faeces		Rotavirus detection in bat saliva	
	n positive/total* (%)	95% CI	n positive/total* (%)	95% CI
***M*. *myotis***	**2/38 (5.2%)**	**[0.6–17.7]**	**3/29 (10.3%)**	**[2.1–27.3]**
***M*. *daubentonii***	**5/18 (27.7%)**	**[9.6–53.4]**	**4/17 (23.5%)**	**[6.8–49.8]**
***M*. *emarginatus***	**2/2 (100.0%)**	**[15.8–100.0]**	**3/8 (37.5%)**	**[8.5–75.5]**
***R*. *ferrumequinum***	**1/6 (16.6%)**	**[0.4–64.1]**	**2/5 (40.0%)**	**[5.2–85.3]**
*M*. *nattereri*	0/9 (0.0%)	[0.0–33.6]	0/4 (0.0%)	[0.0–60.2]
*M*. *beschteini*	0/3 (0.0%)	[0.0–70.7]	0/2 (0.0%)	[0.0–84.1]
*M*. *mystascinus*	0/3 (0.0%)	[0.0–70.7]	0/6 (0.0%)	[0.0–45.9]
*P*. *auritus*	0/1 (0.0%)	[0.0–97.5]	0/6 (0.0%)	[0.0–45.9]
*B*. *barbastellus*	0/1 (0.0%)	[0.0–97.5]	**1/6 (16.6%)**	**[0.4–64.1]**
*M*. *alcathoe*	0/1 (0.0%)	[0.0–97.5]	0/1 (0.0%)	[0.0–97.5]
*R*. *hipposideros*	0/2 (0.0%)	[0.0–84.1]	0/1 (0.0%)	[0.0–97.5]
Total	**10/84 (11.9%)**	**[5.8–20.8]**	**13/85 (15.2%)**	**[8.4–24.7]**

Abbreviations: 95% CI: 95% confidence interval; /: not tested;

* number of tested samples corresponds to amplifiable samples excluding non-interpretable samples

### 3.2. Phylogenetic and BLAST analysis of CoV sequences

**Fig 3:**
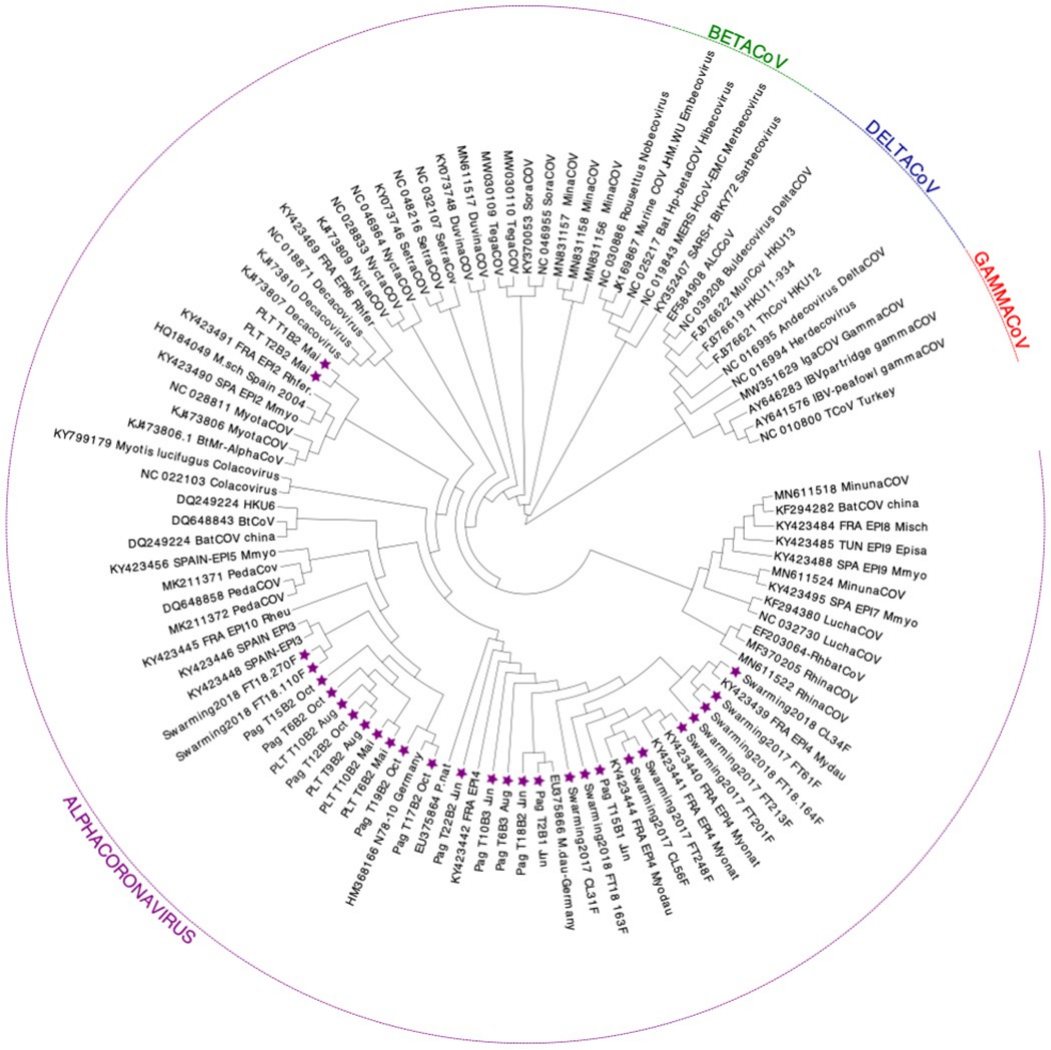
Phylogenetic tree of the coronavirus (CoV) RdRp gene (-438 bp), including 63 referenced sequences and 28 sequences amplified from 28 bat faecal samples. All 28 samples belong to the genus Alphacoronavirus. Sequences acquired in this study are indicated with a purple star. **CHI**: China, **HK**: Hong Kong, **CAN**: Canada, **ENG**: England, **KEN**: Kenya, **USA**: United States of Amercia, **ITA**: Italy, **FRA**: France, **TUN**: Tunisia, **SPA**: Spain, **MOR**: Morocco, **GER**: Germany. **Rh.sp.**: *Rhinolophus sp*., **Rh.**: *Rhinolphus*, **Rh.eu.**: Rhinolophus aegyptiacus, **Rh.eu.**: *Rhinolophus euryale*, **Rh.aff.**: *Rhinolophus affinis*, **M.sch.**: *Miniopterus schreibersii*, **M.dau.**: *Myotis daubentonii*, **M.natt.**: *Myotis nattereri*, **M.m.**: *Myotis myotis*, Rh.ferr.: *Rhinolophus ferrumequinum*, **E.isa.**: *Eptesicus isabellinus*, **H.pr.**: *Hipposideros pratti*, **M.ema.**: *Myotis emarginatus*, **M.l.**: *Myotis lucifugus*, **M.sp.**: *Myotis sp*., **H.vitt.**: *Hipposideros vittatus*, **M.pu.**: *Miniopterus pusillus*, **N.ve.**: *Nyctalus velutinus*, **P.kuh.**: *Pipistrellus kuhlii*, **P.natt.**: *Pipistrellus nathusii*, **M.rick.**: *Myotis ricketti*, **F.ca.**: *Felis catus*, **Sc.kuh.**: *Scotophilus kuhlii*, **Tr.af.**: *Triaenops afer*, **S.ara.**: *Sorex Araneus*.

The phylogenetic tree presented in Fig 3 including 63 referenced CoV sequences representing the four coronavirus genera and 28 sequences from this study, showed that all 28 faecal samples belonged to the *Alphacoronavirus* genus. BLAST results showed a strong similarity with alphacoronaviruses: 100% of nucleotide identity with the α-CoV sequence KY423440.1 (*M*. *nattereri*, France), followed by >96% nucleotide identity with *M*. *daubentonii* (KY423442.1, KY423442.1, and MG923567.2).

### 3.3. Detection of *Leptospira* DNA

A total of 490 faecal samples, 12 urine, 109 lung and 157 kidney were analysed for the presence of the housekeeping DNA gene. β-actin DNA was not detected in 64 faecal samples, 2 urine samples, 51 lung samples and 73 kidney samples, and were consequently considered as non-amplifiable samples. Amplifiable and non-amplifiable samples detected in the different sampling protocols are described in Table 1.A2.

For *Leptospira* assessment, three urine samples collected in the two maternity colonies (30.0% 95CI [6.6–65.2]) tested positive, whereas no *Leptospira* DNA was found in faecal, lung or kidney samples.

#### 3.3.1. Study of two maternity colonies

*3*.*3*.*1*.*1*. *Detection of β-*actin *DNA*. A total of 490 faecal and 12 urine samples were analysed for the presence of β-actin DNA. For faecal samples, 64 (13.0% 95CI [10.2–16.3]) were identified as non-amplifiable samples and 2 (16.6% 95CI [1.5–38.4]) for urine samples. No significant differences were detected when comparing the proportions of non-amplifiable samples in the different biological matrices (faeces and urine) at each site. When considering faecal samples only, the proportion of non-amplifiable samples was significantly higher at Site I (17.0% 95CI [13.1–21.5]) than at Site II (5.0% 95CI [2.1–9.5]) according to the Pearson’s chi-squared test (p*χ*^2^ = 0.0002).

*3*.*3*.*1*.*2*. *Detection of* Leptospira *DNA*. Of the 426 faecal (amplifiable samples, non-interpretable samples excluded), none tested positive for *Leptospira* using real-time PCR whereas 3 out of the 10 urine samples tested positive (30.0% 95CI [6.6–65.2]). These three samples were also positive in conventional PCR (331bp) for *Leptospira*. Species identification showed 100% nucleotide identity with a published sequence of *Leptospira borgpetersenii* CP047520.1. Two samples were collected at Site I harbouring mainly *M*. *daubentonii*, and one sample was collected at Site II harbouring mainly *M*. *myotis*.

#### 3.3.2. Collection of kidneys and lungs from bat carcasses

*3*.*3*.*2*.*1 Detection of β*-actin *DNA*. Of 109 lung and 157 kidney samples collected on carcasses and analysed for the presence of β-actin DNA, 51 lung (46.7% 95CI [37.1–56.5]) and 73 kidney samples (46.4% 95CI [38.5–54.6]) were considered as non-amplifiable samples (Table 1.A2). The proportion of non-amplifiable samples differed significantly between lung and kidney samples compared with faecal samples collected at Site I (p*χ*^2^ = 1.91E-18). Similarly, the proportion of non-amplifiable samples differed significantly between lung or kidney samples and the faecal samples collected at Site II (p*χ*^2^ = 2.60E-22). The proportion of non-amplifiable samples was higher in organs (≈ 46.6%95CI [40.5–52.8]) than in faeces (17.0% 95CI [13.1–21.5] at Site I and 5.0% 95CI [2.1–9.5] at Site II).

*3*.*3*.*2*.*2*. *Detection of* Leptospira *DNA*. Of the 58 lung and 84 kidney samples, none tested positive for *Leptospira* (Table 2).

## 4. Discussion

In recent years, bats have been the subject of numerous studies, not just due to their essential role in ecosystem ecology, but also for public health issues, because bats naturally harbour many different families of viruses, most of which can infect mammals and, to a lesser extent, may be at the origin of serious human diseases [49, 50]. Because bats are hosts to naturally zoonotic viruses, it is paramount to investigate them to assess the risk of spill-over transmission to animals and humans. In this regard, we carried out a study in autochthonous bat species present in France, from different types of biological samples, by targeting diverse viral families with faecal-oral transmission, in addition to lyssavirus commonly detected in bats, as well as *Leptospira*.

The first step of this study was to test all collected samples (faeces, oropharyngeal swabs, urines, kidneys and lungs) for the presence of an endogenous positive control by amplifying β-actin DNA or RNA to detect PCR inhibition. Faecal samples are notoriously difficult to analyse using molecular methods due to the presence of numerous faecal inhibitors [41, 42, 51]. Bile salts, haemoglobin and polysaccharides have also been identified as substances that inhibit PCR, in addition to the reverse transcriptase enzyme, which has been described to be particularly sensitive to inhibitors. Here, the percentage of non-amplifiable samples was high and significant in the three sampling protocols used in this study. These high percentages of non-amplifiable samples can be attributed to the presence of high amounts of inhibitors in the samples. Our investigations showed that the proportions of non-amplifiable faecal samples varied with the sampling method.

PCR inhibition was less frequent at Site II (homemade trap + plastic tarp) than at Site I (only plastic film) for both molecular biology techniques (β-actin DNA and β-actin RNA). These findings can be explained by the presence of the homemade trap at Site II used to prevent flooding of the sampling area and thus limit the degradation of the faecal samples. However, we cannot not exclude the possibility that these differences are due to other factors varying between Site I and Site II, such as bat species or environmental factors.

In addition, the proportions of non-amplifiable oropharyngeal swab samples collected during swarming was higher in 2018 than 2017. This difference can be attributed to the difference in storage buffer: oropharyngeal swabs collected in 2018 were stored in DMEM (n = 36), whereas the oropharyngeal swabs collected in 2017 were stored in RNA Later (n = 194). We noticed that the proportions of non-amplifiable samples were lower in oropharyngeal swab samples stored in DMEM (13.8% of non-exploitable samples) than in samples stored in RNA Later (60.8% of non-amplifiable samples). Although this difference may be due to other environmental factors that differed between 2017 and 2018, it can easily be explained by the type of storage buffer (DMEM or RNA Later). RNA Later, which rapidly penetrates the tissues to stabilize and protect cellular RNA, may act as RNA extraction inhibitors, interfering during the lysis step of the extraction process, leading to a decrease in the efficiency of nucleic acid extraction. Further investigations are needed to conclude on this point.

In our study, the comparison of proportions of non-amplifiable samples between organs (kidneys and lungs) and faecal samples showed that organs contain inhibitors more frequently than faeces. We hypothesize that this difference is due to the long-term storage of carcasses in freezers or due to successive freeze-thaw cycles. Again, this hypothesis must be confirmed by further analysis and comparisons.

To our knowledge, most studies do not mention exogenous or endogenous internal controls validating all the extraction steps (in particular, for nucleic acid extraction) and processing of samples. Of the 15 studies published in France, Germany, Serbia, Italy, USA, Canada, Zambia, Thailand, Grenada, and New Orleans, only 3 studies use an internal positive control for PCR [39, 52, 53]. In 2021, Peterson and Seidlova used an exogenous positive control, but Ayral in 2016 used an endogenous positive control. To our knowledge, many studies do not mention exogenous or endogenous internal controls validating all the extraction steps (in particular, for nucleic acid extraction) and processing of samples. We reviewed the literature on the website NCBI by searching the following keywords: pathogens (i.e. Coronavirus, hepatitis E, Adenovirus, Rotavirus, Lyssavirus, Leptospira and SARS-CoV-2), sample tested (i.e. faeces, oropharyngeal swabs, blood) and the animal tested (i.e. bats, rodents, hedgehogs, pigs, ferrets) Of 15 selected studies published by different authors and in different countries (i.e. France, Germany, Serbia, Italy, USA, Canada, Zambia, Thailand, Grenada New Orleans), only 3 studies reported the use of an internal positive control for PCR. In 2021, Peterson and Seidlova used an exogenous positive control, but Ayral in 2016 used an endogenous positive control. The absence of an internal positive control in PCR can lead to a bias in the interpretation of the results. Many animal sample matrices, such as faeces, saliva and tissues, contain nucleic acid (RNA/DNA) inhibitors as demonstrated in this study and can lead to false negative results. False negatives can lead to erroneous estimations of prevalence in studies based on the presence of pathogens in biological samples. Our study highlights the importance of verifying a sample’s exploitability by amplifying a housekeeping gene such as β-actin. In the absence of this verification step, PCR results can be heavily biased, especially when estimated pathogen prevalence is low.

To our knowledge, this is the first study describing the presence of *Leptospira* DNA in autochthonous bats (*M*. *daubentonii* and *M*. *myotis*) in France. α-CoV and rotavirus have previously been described in France [6, 36, 54] and in Europe [11, 37, 55]; here, we confirmed the presence of α-CoV and rotavirus in four bat species (*M*. *myotis*, *M*. *daubentonii*, *M*. *emarginatus* and *R*. *ferrumequinum*), whereas only rotavirus was detected in *B*. *barbastellus*.

In this study, the prevalence of positive α-CoV samples was higher in faecal samples than in oropharyngeal swab samples. This higher prevalence in faecal samples corroborates previous studies, suggesting that faeces are the best biological matrix for α-CoV detection in bats [11, 37, 56, 57].

In Europe, α-CoV was previously described in different bat species from faecal and/or anal swab samples. For example, α-CoV RNA has been reported in *B*. *barbastellus*, *M*. *myotis*, *Eptesicus serotinus* [58–60] and *Myotis nattereri* bats [61, 62]. Our results (α-CoV prevalence ~22%) are in accordance with previously published studies describing a prevalence of α-CoV RNA ranging from 9% to 75% in various bat species [61, 62]. A published study by Joffrin et al. 2022, revealed an extreme variation of the detection rate of bats shedding viruses over the birthing season (from 0% to 80%) from faecal samples collected during two consecutive years.

In France, three studies have detected the presence of coronavirus RNA in bat populations [6, 36, 54]. Goffard et al. demonstrated the presence of α-CoV in *Pipistrellus* faecal samples with a prevalence of around 4.2% [54]. Monchatre-Leroy et al., based on cadaver intestine analysis, showed the presence of α-CoV RNA in 4 bat species, *P*. *pipistrellus*, *M*. *emarginatus*, *M*. *nattereri* and *Miniopterus schreibersii* [36]. Gouilh et al. 2018 [6], based on faecal analysis on a wider range of species, reported a total of 212 (13.6%) positive samples out of 1551 for α- and β-CoV. α-CoV (9 species called Epi2-10) and β-CoV (1 species called β, Epi 1) were detected in 11 bat species in France, Spain and North-Africa, respectively with β-CoV detected in *Rhinolophus ferrumequinum* only [6]. Prevalence ranged from 8.8% for α-CoV in *M*. *nattereri* to 37.9% for β-CoV in *R*. *ferrumequinum*.

In the present study, in which only the genus *Alphacoronavirus* was screened for, we observed the presence of α-CoV RNA in four bat species (*M*. *myotis*, *M*. *daubentonii*, *M*. *emarginatus* and *R*. *ferrumequinum*), all species in which there are reports of α-CoV in France; we did not detect α-CoV RNA in *P*. *pipistrellus* bats because this species was not part of our samples collected during swarming. Therefore, it is important carry out more studies with sufficiently large numbers of samples and bat species to better understand the diversity and the distribution of coronaviruses among French bat populations.

Temporal variation in the presence of α-CoV excretion was observed at Site I and II. Interestingly, a peak of α-CoV RNA was observed for the September-October period at Site I (*M*. *daubentonii* maternity colony) and for the August-October period at Site II (*M*. *myotis* maternity colony). Between May and June, the maternity colony is comprised of female bats ready to give birth, whereas in the autumn (September–October), the bat colony is larger due to the presence of juveniles. At both sites, the presence of α-CoV seemed to increase after the parturition period. Thus, the presence of juvenile bats may explain the peaks of α-CoV detection in September–October at Site I and in August at Site II. Drexler et al. [11] also showed an amplification peak of CoV and astrovirus after parturition was associated with the presence of newborn bats that had not yet developed their own adaptative immunity. Several other studies have also reported increased circulation of CoV in young bats around the world [63–65].

The presence of rotavirus RNA has been previously described in faecal samples of *Rousettus aegyptiacus* in Kenya [66], and of *M*. *schreibersii* in Serbia [38], as well in carcasses of *M*. *mystacinus* in France [9]. Our study suggests the presence of partial rotavirus RNA in *M*. *myotis*, *M*. *daubentonii*, *M*. *emarginatus*, *R*. *ferrumequinum* and *B*. *barbastellus* with 13 real-time RT-PCR positive oropharyngeal swabs collected during the swarming period and two positive faecal samples collected during study of maternity colonies. Interestingly, the prevalence of positive rotavirus samples was higher in oropharyngeal swab samples than in faecal samples, indicating that saliva is the best biological matrix for rotavirus detection in bats in our study.

However, the positive oropharyngeal swab samples revealed using real-time RT-PCR targeting the *nsp3* gene of *Rotavirus A* tested negative using conventional RT-PCR with primers targeting the *nsp4* gene of *Rotavirus A* in rabbits [48], limiting the study. This discrepancy can be explained either by the low positivity observed for these samples, the positive samples showing Ct values varying between 27 and 32, or by the repeated freeze/thaw cycles of RNA extracts causing potential partial RNA degradation. Another hypothesis may be the high diversity of the *nsp4* gene in *Rotavirus A*. Moreover, Dacheux et al., [9] showed that a new specimen of *Rotavirus A* isolated from a carcass of *M*. *mystacinus* in France was more closely related to group A rotaviruses than groups B, C and D, albeit with a sequence found at a basal position in the phylogenetic tree, very distant to the group constituted of sequences representative of equine, caprine, human, simian or avian *Rotavirus A* [9]. Sasaki et al. (2018) [67] lends support to the hypothesis of a *Rotavirus A* genotype specific to bats with a distinct lineage of *nsp4* in bats: they demonstrated that a positive sample of their study showed only <80% nucleotide sequence identity in the *nsp4* gene with all available sequences in public databases [67].

To date, bats have been reported as reservoirs of 15 of the 17 known lyssavirus species. Five lyssavirus species have been isolated in bats in Europe, namely European bat 1 lyssavirus (EBLV-1), European bat 2 lyssavirus (EBLV-2), Bokeloh bat lyssavirus (BBLV), Lleida bat lyssavirus (LLEBV) and Kotalahti bat lyssavirus (KBLV). In Europe, these bat lyssaviruses have been reported in specific bat hosts: *Eptesicus serotinus* and *Eptesicus isabellinus* for EBLV-1, *M*. *daubentonii* and *Myotis dasycneme* for EBLV-2, *M*. *nattereri* for BBLV, *Miniopterus schreibersii* for LLEBV, and *Myotis brandtii* for KBLV [68]. EBLV-1 is the main lyssavirus species encountered in metropolitan France, followed by a few isolations of BBLV and LLEBV [69–73]. Begeman et al. 2020 reported the presence of lyssavirus RNA using real-time RT-PCR in faecal pellets (6/7 bats positive) and in oral swabs (with 7/7 bats positive) of seven *E*. *serotinus* that were positive for European bat lyssavirus in the brain [74]. In our study, no sample tested positive for lyssavirus RNA. However, the bat species in this study (*M*. *myotis* and *M*. *daubentonii)* have never tested positive for bat rabies in France. Moreover, previous published results showed the quasi-absence of lyssavirus RNA in oropharyngeal samples of lyssavirus host species [75–78]. Bat lyssaviruses can indeed persist despite a low prevalence of infection [79] and the probability of detecting lyssavirus RNA in saliva is therefore very low.

In our study, no sample tested positive for CDV RNA. To our knowledge, no studies on the CDV virus have been carried out in bats, although Paramyxoviruses are one of the most screened viruses in fruit and insectivorous bats, as shown by the dbatvir database. To date, the genus *Morbillivirus* (family Paramyxoviridae), is composed by different species including *Measles morbillivirus* (MeV), *Canine morbillivirus*, *Rinderpest morbillivirus* (RPV), *Cetacean morbillivirus*, *Feline morbillivirus*, *Phocine morbillivirus* and *Small ruminant morbillivirus* and a novel morbillivirus species in a Brazilian vespertilionid bat species (*Myotis riparius)*, called myotis bat morbillivirus (MBaMV) [80]. Other *Morbillivirus-related* viruses have been detected in Germany in insectivorous bats [81], as well as in in the Comoros Islands, Mauritius and Madagascar [82]. We investigated the presence of CDV in bats on the hypothesis of a spillover from a wild animal (such red fox) naturally infected by CDV on bats. Indeed, this virus which causes a fatal disease mainly in dogs has been reported in several field species such as wild canids, procyonids, ailurids, mustelids, viverrids, hyenas, or even lions [83]. In addition, during some CDV outbreaks, land carnivores (dogs and wolves), have been suspected of being vectors of the infectious agent. Moreover, lethal infections have been described in non-carnivore species as well on non-human primates, demonstrating the remarkable capacity of the pathogen to cross species barriers. This crossing of the interspecies barrier of CDV between domestic and wildlife animals underscores the importance of investigating the presence of this virus in bats, which are among the most zoonotic disease harboring wildlife animals. Because of the negative results we observed in our study, it would be interesting to expand our research to all species of the Morbillivirus genus, using a more broadly reactive PCR assay (RMH system) [84].

In 2021, Seidlova et al. [39], demonstrated the presence of *Leptospira interrogans* in urine samples of Paleartic bats. In our study, we tested different bat matrices for *Leptospira*, bat faecal and urine samples from two maternity colonie, kidney and lung from bat cadavers. Only urine samples tested positive for *Leptospira*. The Sanger sequences of the positive samples showed strong genetic identity with previously described *L*. *borgpetersenii* sequences. Although *Leptospira* is known to be detected in rat kidney and lung samples [85], we did not detect *Leptospira* in carcasses. One of the explanations, other than the absence of these pathogens, may be the long storage of bat cadavers at <-65°C associated with numerous freeze-thaw cycles that may have degraded the DNA. The detection of *Leptospira* DNA in bat urine and not in faeces or kidney or lung tissue makes urine as the best biological matrix for detecting *Leptospira* in bats in our study.

## 5. Conclusion

This study underlines the importance of screening the presence of PCR inhibitors before performing any molecular, epidemiological analysis, as well for interpreting PCR results in bats. Furthermore, the proportion of inhibited reactions can vary according to the methodology employed, particularly with regards to storage and sampling. The present exploratory study also showed the presence of *Leptospira* DNA in autochthonous bats in France, in addition to α-CoV and rotavirus RNA, already described in European bat populations. These two RNA pathogens (rotavirus and α-CoV) and *Leptospira* DNA were detected in both *M*. *myotis* and *M*. *daubentonii* and both α-CoV and rotavirus were detected in *M*. *emarginatus* and *R*. *ferrumequinum*. Rotavirus was also detected in *B*. *barbastellus*. Temporal variation in the detection of α-CoV was also observed, with higher frequencies in late summer and in October, suggesting that juveniles potentially play an important role in the dynamics of these viruses. This study constitutes the basis for future epidemiological studies in which the effect of seasonality over the long term can be more thoroughly investigated. Analysis of ground stool samples of bat colonies, which represents an ideal non-invasive method to investigate pathogens in bats, can help to better determine the role bats play in the spread of zoonotic infections. Understanding the ecology of bat-borne pathogens can indeed help mitigate the emergence of zoonotic disease outbreaks.

## Supporting information

S1 File (DOCX)

S2 File (XLSX)

S3 File (XLS)

## Abbreviations

Alphacoronavirus: α-CoV
CDV: Canine Distemper Virus
DNA: Deoxyribonucleic acid
PCR: Polymerase Chain Reaction
RdRp: RNA-dependent RNA polymerase
RNA: Ribonucleic acid
