# Supplementary material for: Assessment of virus and Leptospira carriage in bats in France
Source: PLoS One. 2023 Oct 20;18(10):e0292840. doi: 10.1371/journal.pone.0292840 (PMC10588846; doi:10.1371/journal.pone.0292840)
Supplement: S1 File — (DOCX) [file pone.0292840.s001.docx]

1. **Detection of lyssavirus, alphacoronavirus, rotavirus and canine distemper virus RNA**

**1.1. RNA virus extraction**

B

Faecal samples: bat faeces were freshly prepared before RNA extraction, as follows. Each faecal pellet was ground with a volume of 120 µL of 1X PBS buffer (Sigma-Aldrich, Saint Quentin-Fallavier, France) then centrifuged for 5 min at 30,000 x g.

RNA extraction was performed on 50 µL of the supernatant using the ZymoBIOMICS RNA Minikit (Ozyme, Saint-Cyr-l’École, France), following the manufacturer’s recommendations. RNA was eluted in 100 µL of elution buffer (ZymoBIOMICS RNA Minikit) and stored at < -65°C.

Oropharyngeal swabs: RNA was extracted with the QIAmp Viral RNA Mini Kit (Qiagen, Courtaboeuf, France) from 140 µL of suspension and eluted in 60 µL of the Qiagen elution buffer. RNA extraction was performed according to the manufacturer’s instructions. RNAs were stored at <-65°C.

**1.2. Real-time RT-PCR**

All primers and probes used for the detection of RNA viruses are described in S1 Table.

**1.2.1. SYBR Green β-actin RT-PCR**

The housekeeping gene β-actin was amplified for each tested sample with SYBR Green RT-PCR under the same conditions, using primers previously described in Wakeley et al. [1].

The SYBR Green β-actin RT-PCR was performed in a final volume of 25 µL containing 2.5 µL of RNA, 12.5 µL of Quantitect SYBR Green Master Mix (Quantitect SYBR Green RT-PCR Kit, Qiagen), 0.75 µL of each primer ß Act intronic and ß Act reverse (20 µM each) and 0.25 µL of Quantitect RT Mix. The PCR was performed with the following conditions: 3 min at 50°C, 15 min at 95°C, 45 cycles of 30s at 94°C, 30s at 55°C, 30s at 72°C and followed by a final step of Melt 5 s/step, 65-95°C.

**1.2.2. SYBR Green lyssavirus RT-PCR**

The detection of lyssavirus RNA was performed with pan-lyssavirus primers previously described in Wakeley et al. [1]. Pan-lyssavirus primers allow the specific detection of all known lyssavirus RNA.

The SYBR Green lyssavirus RT-PCR was performed in a final volume of 25 µL containing 2.5 µL of RNA, 12.5 µL of Quantitect SYBR Green Master Mix (Quantitect SYBR Green RT-PCR Kit), 0.75 µL of each primer JW12 and N165-146 (20 µM) and 0.25 µL of Quantitect RT Mix. The PCR was performed with the following conditions: 3 min at 50°C, 15 min at 95°C, 45 cycles of 30s at 94°C, 30s at 55°C, 30s at 72°C and followed by a final step of Melt 5 s/step, 65-95°C.

**1.2.3. TaqMan canine distemper virus RT-PCR**

A two-step RT-PCR was performed for the detection of CDV RNA, as follows.

**1.2.3.1. Reverse transcription: generation of cDNA**

Two µL of extracted RNA were used as a template to generate cDNA with a commercial kit Reverse Transcriptase Core kit (Eurogentec, Angers, France). The reaction was performed in a final volume of 20 µL containing 2 µL of RNA, 2 µL of 10X reaction buffer, 4 µL of MgCl_2_ (25 mM), 4 µL of dNTP (2.5 mM), 1 µL of random nonamer, 0.4 µL of RNase inhibitor (20 U/µL), 0.4 µL of DMSO (Sigma-Aldrich, Lezennes, France) and 0.5 µL of Euroscript RT enzyme. The reaction was performed with following conditions: 10 min at 25°C, 30 min at 48°C followed by a final step of 5 min at 95°C. The cDNA was stored at -65°C.

**1.2.3.2. TaqMan PCR**

TaqMan Real-Time PCR amplification was performed in 20 µL reaction volume containing 2 µL of cDNA, 10 µL of Takyon Master Mix (Takyon^TM^ ROX Probe 2X Master Mix dTTP, Angers, France), 0.3 µL of each primer CDV-F and CDV-R (20 µM) and 0.2 µL of probe CDV-P (20 µM). The PCR was performed with the following conditions: 2 min at 50°C, 40 cycles of 3 min at 95°C, 10 s at 95°C and followed by a final step of 60 s at 60°C.

**1.2.4. SYBR Green Rotavirus A RT-PCR**

A two-step RT-PCR was performed for the detection of *Alphacoronavirus* and *Rotavirus A* RNA, as follows.

**1.2.4.1. Reverse transcription: generation of cDNA**

Two µL of extracted RNA were used as a template to generate cDNA with a commercial kit Reverse Transcriptase Core kit (Eurogentec). The reaction was performed in a final volume of 20 µL containing 2 µL of RNA, 2 µL of 10X reaction buffer, 4 µL of MgCl_2_ (25 mM), 4 µL of dNTPs (2.5 mM), 1 µL of random nonamer, 0.4 µL of RNase inhibitor (20 U/µL), 0.4 µL of DMSO (Sigma-Aldrich) and 0.5 µL of Euroscript RT enzyme. The reaction was performed with following conditions: 10 min at 25°C, 30 min at 48°C following with a final step of 5 min at 95°C. The cDNA was stored at -65°C.

**1.2.4.2. SYBR Green *Alphacoronavirus* PCR**

SYBR Green Real-Time PCR amplification was performed in 20 µL reaction volume containing 2.5 µL of cDNA, 10 µL of Takyon Master Mix (Takyon ^TM^ No ROX SYBR 2X Master Mix blue dTTP). 0.3 µL of each primer CoV-F1, CoV F2 (20 µM) and 0,6 µL of CoV-R (20 µM). The PCR was performed with the following conditions: 2 min at 50°C, 3 min at 95°C, 40 cycles of 10s at 95°C, 30s at 62°C followed by a final step of Melt 5 s/step, 65-95°C.

**1.2.4.3. SYBR Green *Rotavirus A* PCR**

The *Rotavirus A* RNA detection was performed with primers targeting the rotavirus *nsp3* gene, previously described by Zeng et al. (2008) for the specific detection of *Rotavirus A* RNA.

The SYBR Green PCR was performed in a final volume of 20 µL containing 2.5 µL of cDNA, 10 µL of Takyon Master Mix (Takyon ^TM^ No ROX SYBR 2X Master Mix blue dTTP), 0.3 µL of each primer ROT-F (20 µM) and ROT-R (20 µM). The PCR was performed with the following conditions: 2 min at 50°C, 3 min at 95°C, 40 cycles of 10s at 95°C, 45s at 56°C followed by a final step of Melt 5 s/step, 65-95°C.

**1.3. Conventional RT-PCR**

A conventional pan-CoV RT-PCR was performed for the specific detection of coronavirus RNA.

S2 Table summarizes all primers used for the detection of coronavirus RNA using conventional RT-PCR.

**1.3.1. Reverse Transcription**

Five µL of extracted RNA were used as template to generate cDNA with the RT Maxima H Minus kit (ThermoFisher Scientific, Dardilly, France). The reaction was performed in a 20 µL volume containing 5 µL of RNA, 0,25 µL of hexanucleotides primers (0.2 µg/µL) (Sigma-Aldrich, Saint Quentin-Fallavier, France), 1 µL of dNTP (10mM), 4µL of RT Buffer 5X, 0,5 µL of RNase OUT (40U/µL) (Sigma-Aldrich, Saint Quentin-Fallavier, France), 0.5 µL of DMSO (Sigma-Aldrich, Lezennes, France) and 1 µL of Maxima RTase enzyme (200U/µL). The reaction was performed with the following conditions: 10 min at 25°C, 30 min at 50°C followed by a final step of 5 min at 85°C. cDNA was stored at below -65°C.

**1.3.2. *Alphacoronavirus* RNA detection (pan-CoV RT-PCR)**

Three µL of cDNA was used as template for the detection of a portion of the RdRp coding region giving PCR products of 438 bp). We used primers BatCoV pol 15197 and Bat- CoV pol 15635 previously described by described in Woo et al. (2005) and slightly modified by Gouilh et al. (2011). Primers were designed from the multiple alignment of the nucleotide sequences of available polymerase genes of known coronaviruses allowing the detection of all coronaviruses. PCR amplification was performed in a 25 µL reaction volume containing 3 µL of cDNA, 2.5 µL of 10X PCR Buffer without MgCl_2_ (Invitrogen, Marseille, France), 0.75 µL of 50 mM MgCl_2_, 1 µL of dNTPs (10 mM each) and 0.5 µL of Taq DNA polymerase (5 U) (Invitrogen, Marseille, France) and 1 µL of each primer (20 µM). The PCR was performed with the following conditions: 2 min at 94°C, 11 cycles of 30s at 94°C, a 1°C touch down decrease of the annealing temperature from 60° to 50°C, 90s at 72°C, then 40 cycles of 30 s at 94°C, 45 s at 50°C and 90 s at 72°C and followed by a final extension step of 10 min at 72°C.

**1.4. Reverse transcription, PCR and Real-Time RT-PCR control:**

For each RT, PCR or RT-PCR, a positive and a negative control were used.

For β-actin detection, a positive control was used in real-time PCR (RNA extracted from bird brain tissue sample).

For lyssavirus detection, a positive control was used in real-time PCR (RNA extracted from brain tissue sample from a bat that tested positive for rabies).

For CDV detection, synthetic RNA, generated by *in vitro* transcription (TranscriptAid T7 High Yield Transcription Kit, ThermoFisher Scientific, Dardilly, France) from the oligonucleotide extremer (5’-GCATTAATTCTCCGGAAAACTCATGCAACCCAAGAGCCGGA-TACATAGTTTCAATGCCAAACTTGATAGTTAGGATGAAACTAGCTAGCGGCCCTATAGTGAGTCGTATTA-3’), was used in real-time PCRs as a positive control at the limit of detection (threshold set between 1 and 100x of the LD method).

For *Alphacoronavirus* detection, a synthetic RNA, generated by *in vitro* transcription (TranscriptAid T7 High Yield Transcription Kit, ThermoFisher Scientific, Dardilly, France) from the oligonucleotide extremer (5’- GACATTGGCACTTACAGCCTGAAATATGTTAAAGACAGAATT-GGCATAGGCAGTAGTAGCATCACCTGACGTGGCCCTATAGTGAGTCGTATTA -3’), was used in real-time PCRs as a positive control at the limit of detection (threshold set between 1 and 100x of the LD method).

For rotavirus detection, synthetic RNA, generated by *in vitro* transcription (TranscriptAid T7 High Yield Transcription Kit, ThermoFisher Scientific, Dardilly, France) from the oligonucleotide extremer (5’-CAACAGAATCATCAACTTCAACCTCACCTCTAGCCAATTTCACGCGCATGTTTTGAT-CAATTCCTTTTGCTGATAACATCATACGTCCCTATAGTGAGTCGTATTACA-3’), was used in real-time PCRs as positive control at the limit of detection (LD) (threshold set between 1 and 100x of the LD method).

A negative RT control (assay without reverse transcriptase) was included in each assay to check for non-specific amplification.

A negative PCR control was included in each assay using water (RNase- and DNase-free) (ThermoFisher Scientific, Illkirch-Graffenstaden, France).

1. **Detection of *Leptospira* DNA**
   1. **DNA extraction**

Tissue and urine samples: a pre-extraction step was carried out to prepare kidneys and lungs. Twenty mg of each organ was mixed with 180 µL of lysis buffer T1 (Macherey Nagel, Hoerdt, France) and 20 µL of Proteinase K (Macherey Nagel). Samples were vortexed and then centrifuged for 30 s at 4000 x g. All the lysates were used for the next step of extraction. DNA extraction was performed using the Magvet MV384 (ThermoFisher Scientific, Villebon-sur-Yvette, France), following the manufacturer’s recommendations. DNA was eluted in 80 µL of elution buffer (Magvet MV384) and stored at -20°C.

Faecal samples: DNA extraction was performed from 50 µL of tissue supernatant prepared as described in section 1.1. DNA extraction was performed using the Nucleospin DNA stool kit (Macherey Nagel), following the manufacturer’s recommendations. DNA was eluted in 100 µL of elution buffer (Nucleospin DNA Stool) and stored at -20°C until analysis.

- 1. **TaqMan β-actin PCR**

A TaqMan β-actin PCR was performed in a final volume of 25 µL containing 4 µL of DNA, 12.5 µL 2XRT-PCR Buffer (AgPath One-Step RT-PCR, ThermoFisher scientific, Dardilly, France), 2.5 µL of each primer Actinas1 and Actinas2 and ActinPr1 (4 µM). The PCR was performed with the following conditions: 10 min at 95°C, 40 cycles of 15 s at 95°C, and followed by a final step of 60 s at 60°C.

- 1. **TaqMan *Leptospira* PCR**

Primers used for the detection of leptospires for real-time PCR are described in S1 Table.

*Leptospira* PCR targeted a partial 16S RNA gene and is specific to pathogenic *Leptospira* [2]. TaqMan real-time PCR amplification was performed in a reaction volume of 25 µL containing 4 µL of DNA, 12.5 µL of 2XRT-PCR Buffer (AgPath One-Step RT-PCR, ThermoFisher Scientific, Dardilly, France), 2.5 µL of each primer 16S patho-F and 16S patho-R and 16S patho-P (20 µM). The PCR was performed with the following conditions: 10 min at 95°C, 40 cycles of 15 s at 95°C, and following with a final step of 60 s at 60°C.

- 1. **Conventional *Leptospira* PCR**

Primers used for the detection of leptospires and species identification are described in S1 Table.

A volume of 5 µL of DNA was used as template for amplification of the partial 16S rRNA gene. We used primers previously described by Mérien et al. [3]. PCR amplification was performed in a 50 µL reaction volume containing 5 µL of DNA, 10 µL of 10X PCR Buffer without MgCl_2_ (HotStarTaq DNA polymerase, Qiagen), 2 µL of 50 mM MgCl_2_, 1 µL of dNTPs (10 mM each) and 0.5 µL of HotstarTaq DNA polymerase (5 U/mL) and 1 µL of each primer LEPTA and LEPTB (0.2 µM). The PCR was performed with the following conditions: 15 min at 95°C, 39 cycles of 30 s at 95°C, 30 s at 57°C and 1 min at 72°C and followed by a final step of extension of 10 min at 72°C

- 1. **PCR control**

For TaqMan β-actin detection, a positive control was used (DNA extracted from dog)

For *Leptospira* detection, a positive control was used (DNA extracted from a *Leptospira interrogans* strain)

A negative PCR control was included in each assay using water (RNase- and DNase-free) (ThermoFisher Scientific, Illkirch-Graffenstaden, France).

- 1. **Limit of detection LD_PCR_ and LD_Method_:**

**LD_PCR_ and LD_Method_ were determined by RT-qPCR for each of the following three targets: Canine Distemper Virus, Rotavirus, and Alphacoronavirus.**

**The LD_PCR_ was estimated >75 copies/μL of RNA for Canine Distemper Virus and Rotavirus and it is more than 90 copies/μL of RNA for Alphacoronavirus.**

**The LD_Method_ was estimated to be >100 copies/μL of RNA for all three targets.**

**The LD_PCR_ determined by SYBR Green RT-PCR for Lyssavirus detection was estimated to be 25 copies/μL while the LD_Method_ was estimated to be more than 100 copies/μL. The LD_PCR_ was performed using synthetic GT1 RNA and was estimated at 50 copies/μL. In addition, LD_Method_ was performed using a supernatant from the tissue shred of a chicken brain. The LD_Method_ was estimated to be 100 copies/μL.**

**For Leptospira sensibility, 8 dilutions were realized from bacterial culture 10^^7^bies/ml. All the 8 dilutions were amplified.**

**S1 Table.**  Characteristics of primers and probes used in the real-time PCRs

| Target | Primers | Sequence (5’- 3’) | Polarity | Gene | Reference |
| --- | --- | --- | --- | --- | --- |
| Housekeeping gene: | | | | | |
| β-actin (RNA) | ß Act intron | CGATGAAGATCAAG/ATCATTGC | + | Exons 4, 5 | [1] |
|  | ß Act reverse | AAGCATTTGCGGTGGAC | - |  |  |
| β-actin (DNA) | Actinas1 | CAGCACAATGAAGATCAAGATCATC | + |  | [3] |
|  | ActinPr1 | TCGCTGTCCACCTTCCAGCAGATGT | Probe |  |  |
|  | Actinas 2 | CGGACTCATCGTACTCCTGCTT | - |  |  |
| RNA viruses | | | | | |
| *Alphacoronavirus* | CoV-F1 | TCDGGTGATGCTACYACTGC | + | Rdrp | [4] |
|  | CoV-F2 | TCTGGTGATGCTACTACWGC | + |  |  |
|  | CoV-R | CATTRGCACTTACAGCCTGRAA | - |  |  |
| Rotavirus | ROT-F | ATGATGTTRTCAKCAAAAGG | + | *nsp3* | [5] |
|  | ROT-R | GAATCATCAACTTCAACYTC | - |  |  |
| Lyssavirus | N165-146 | GCAGGGTAYTTRTACTCATA | + | Nucleoprotein | [1] |
|  | JW12 | ATGTAACACCYCTACAATG | - |  |  |
| Canine distemper virus | CDV-F | AGCTAGTTTCATCYTAACTATCAARTT | + | Nucleocapsid protein gene H | [6] |
|  | CDV-P | FAM-ACCCRAGAGCCGGATACAT  AGTTTCAATGC-BHQ1 | Probe |  |  |
|  | CDV-R | TTAAYTCTCCAGAAAACTCATGC | - |  |  |
| Bacterial DNA | | | | | |
| *Leptospira* | 16S patho-F | CGGGAGGCAGCAGTTAAGAA | + | 16S | [2] |
|  | 16S patho-P | HEX- GCRATGTGATGATGGTACCTG CCT- BHQ1 | Probe |  |  |
|  | 16S patho-R | AACAACGCTTGCACCATACG | - |  |  |

Abbreviations: +: forward primer; -: reverse primer

**S2 Table.** Characteristics of primers used in conventional RT-PCR.

| Target | Primers | Sequence 5’ - 3’ | Polarity | Gene | Reference |
| --- | --- | --- | --- | --- | --- |
| Coronavirus | Bat-CoV pol 15197 | GGTTGGGAYTAYCCWAARTGTGA | + | RdRp, *nsp12* coding regions | [7][8] |
|  | Bat-CoV pol 15635 | CCATCRTCMGAHARAATCAT CATA | - |  |  |
| *Leptospira* | LEPTA | GGCGGCGCGTCTTAAACATG | + | 16S | [9] |
|  | LEPTB | TTCCCCCCATTGAGCAAGATT | - |  |  |

Abbreviations: +: forward primer; -: reverse primer

**S3 Table.** Characteristics of the coronavirus (CoV) RdRp gene reference sequences retrieved from GenBank and samples from this study

| No. | Country | Organism | Year | GenBank Accession no. | Source |
| --- | --- | --- | --- | --- | --- |
| 1 | China | Avian CoV | 2019 | MW351629 | [11] |
| 2 | China | Avian CoV | 2003 | AY646283 | / |
| 3 | Canada | Turkey CoV | 2007 | NC_010800 | [12] |
| 4 | China | Avian CoV | 2007 | AY641576 | / |
| 5 | China | Night-heron CoV | 2007 | NC_016994 | [13] |
| 6 | Hong Kong | Wigeon CoV | 2008 | NC_016995 | [13] |
| 7 | Hong Kong | Thrush CoV | 2007 | FJ376621 | [14] |
| 8 | Hong Kong | Bulbul CoV | 2007 | FJ376619 | [14] |
| 9 | Hong Kong | Munia CoV | 2007 | FJ376622 | [14] |
| 10 | Hong Kong | Porcine CoV | 2010 | NC_039208 | [13] |
| 11 | China | Asian leopard cat CoV | 2006 | EF584908 | [15] |
| 12 | Kenya | SARS-CoV | 2007 | KY352407 | [16] |
| 13 | Saudi Arabia | MERS-CoV | 2012 | NC_019843 | [17] |
| 14 | China | Bat CoV | 2013 | NC_025217 | [18] |
| 15 | USA | Murine CoV | 1947 | JX169867 | / |
| 16 | China | Bat CoV | 2014 | NC_030886 | [19] |
| 17 | China | Ferret CoV | 2019 | MN831156 | / |
| 19 | China | Ferret CoV | 2019 | MN831157 | / |
| 21 | China | Shrew CoV | 2014 | KY370053 | [20] |
| 22 | China | Feline CoV | 2018 | MW030110 | / |
| 23 | USA | Feline CoV | 2018 | MW030109 | / |
| 24 | Kenya | Bat CoV | 2018 | MN611517 | [21] |
| 25 | Kenya | Bat CoV | 2010 | KY073748 | [22] |
| 26 | Kenya | Bat CoV | 2010 | NC_032107 | [22] |
| 28 | Kenya | Bat CoV | 2008 | KY073746 | [22] |
| 29 | Italy | Bat CoV | 2015 | NC_046964 | / |
| 31 | China | Bat CoV | 2013 | KJ473809 | [18] |
| 32 | China | Bat CoV | 2013 | MN611522 | [18] |
| 33 | China | Bat CoV | 2017 | MF370205 | [23] |
| 34 | China | Bat CoV | 2006 | EF203064 | [24] |
| 35 | China | Lucheng CoV | 2013 | NC_032730 | [25] |
| 36 | China | Lucheng CoV | 2013 | KF294380 | [25] |
| 37 | Spain | Bat CoV | 2015 | KY423495 | [26] |
| 38 | China | Bat CoV | 2018 | MN611524 | [21] |
| 39 | Spain | Bat CoV | 2011 | KY423488 | [26] |
| 40 | Tunisia | CoV | 2012 | KY423485 | [26] |
| 41 | France | Bat CoV | 2014 | KY423484 | [26] |
| 42 | China | Bat CoV | 2012 | KF294282 | [25] |
| 43 | China | Bat CoV | 2018 | MN611518 | [25] |
| 44 | France | BatCoV | 2014 | KY423469 | [26] |
| 45 | China | Bat CoV | 2005 | NC_018871 | [27] |
| 46 | China | Bat CoV | 2013 | KJ473810 | [18] |
| 47 | China | Bat CoV | 2013 | KJ473807 | [18] |
| 48 | France | Bat CoV | 2015 | KY423491 | [26] |
| 49 | Spain | Bat CoV | 2004 | HQ184049 | [28] |
| 50 | Spain | Bat CoV | 2011 | KY423490 | [26] |
| 52 | China | Bat CoV | 2011 | KJ473806 | [18] |
| 54 | Canada | Bat CoV | 2010 | KY799179 | [29] |
| 56 | China | Bat CoV | 2006 | DQ249224 | [30] |
| 58 | China | Bat CoV | 2006 | DQ249224 | [30] |
| 59 | Spain | Bat CoV | 2015 | KY423456 | [26] |
| 60 | China | Bat CoV | 2017 | MK211371 | [32] |
| 61 | China | Bat CoV | 2005 | DQ648858 | [31] |
| 62 | China | Bat CoV | 2017 | MK211372 | / |
| 63 | France | Bat CoV | 2012 | KY423445 | [26] |
| 65 | Spain | Bat CoV | 2015 | KY423448 | [26] |
| 66 | Germany | Bat CoV | 2008 | HM368166 | [4] |
| 67 | Germany | Bat CoV | 2007 | EU375864 | [33] |
| 68 | France | Bat CoV | 2015 | KY423442 | [26] |
| 69 | Germany | Bat CoV | 2007 | EU375866 | [33] |
| 70 | France | Bat CoV | 2014 | KY423439 | [26] |
| 71 | France | Bat CoV | 2015 | KY423444 | [26] |
| 72 | France | Bat CoV | 2015 | KY423441 | [26] |
| 73 | France | Bat CoV | 2015 | KY423440 | [26] |
| 74 | France | PLT T1B2 Mai | 2019 | MZ893205 | This study |
| 75 | France | PLT T2B2 Mai | 2019 | MZ893206 | This study |
| 76 | France | Swarming2018 FT18.270F | 2019 | MZ893219 | This study |
| 77 | France | Swarming2018 FT18.110F | 2019 | MZ893216 | This study |
| 78 | France | Pag T15B2 Oct | 2019 | MZ893195 | This study |
| 79 | France | Pag T6B2 Oct | 2019 | MZ893201 | This study |
| 80 | France | PLT T10B2 Aug | 2019 | MZ893203 | This study |
| 81 | France | Pag T12B2 Oct | 2019 | MZ893193 | This study |
| 82 | France | PLT T9B2 Aug | 2019 | MZ893208 | This study |
| 83 | France | PLT T10B2 Mai | 2019 | MZ893204 | This study |
| 84 | France | PLT T6B2 Mai | 2019 | MZ893207 | This study |
| 85 | France | Pag T19B2 Oct | 2019 | MZ893198 | This study |
| 86 | France | Pag T17B2 Oct | 2019 | MZ893196 | This study |
| 87 | France | Pag T22B2 Jun | 2019 | MZ893199 | This study |
| 88 | France | Pag T10B3 Jun | 2019 | MZ893192 | This study |
| 89 | France | Pag T6B3 Aug | 2019 | MZ893202 | This study |
| 90 | France | Pag T18B2 Jun | 2019 | MZ893197 | This study |
| 91 | France | Swarming2017 CL31F | 2019 | MZ893209 | This study |
| 92 | France | Swarming2018 FT18 163F | 2019 | MZ893217 | This study |
| 93 | France | Pag T15B1 Jun | 2019 | MZ893194 | This study |
| 94 | France | Swarming2017 CL56F | 2019 | MZ893210 | This study |
| 95 | France | Swarming2017 FT248F | 2019 | MZ893213 | This study |
| 96 | France | Swarming2017 FT201F | 2019 | MZ893211 | This study |
| 97 | France | Swarming2017 FT213F | 2019 | MZ893212 | This study |
| 98 | France | Swarming2018 FT18.164F | 2019 | MZ893218 | This study |
| 99 | France | Swarming2017 FT61F | 2019 | MZ893214 | This study |
| 100 | France | Swarming2018 CL34F | 2019 | MZ893215 | This study |
| 101 | France | Pag T2B1 Jun | 2019 | MZ893200 | This study |

**S4 Table** Details of faecal samples shown positive using conventional pan-coronavirus (CoV) RT-PCR for the presence of CoV RNA

| Samples | Bat species | Collection mode | Year | Region |
| --- | --- | --- | --- | --- |
| PLT T1B2 May | *Myotis myotis* | Study of maternity colonies | 2019 | Lorraine |
| PLT T2B2 May | *Myotis myotis* | Study of maternity colonies | 2019 | Lorraine |
| Swarming2018 FT18.270F | *Myotis myotis* | Swarming | 2018 | Brittany |
| Swarming2018 FT18.110F | *Myotis myotis* | Swarming | 2018 | Brittany |
| Pag T15B2 Oct | *Myotis daubentonii* | Study of maternity colonies | 2019 | Lorraine |
| Pag T6B2 Oct | *Myotis daubentonii* | Study of maternity colonies | 2019 | Lorraine |
| PLT T10B2 Aug | *Myotis myotis* | Study of maternity colonies | 2019 | Lorraine |
| Pag T12B2 Oct | *Myotis daubentonii* | Study of maternity colonies | 2019 | Lorraine |
| PLT T9B2 Aug | *Myotis myotis* | Study of maternity colonies | 2019 | Lorraine |
| PLT T10B2 May | *Myotis myotis* | Study of maternity colonies | 2019 | Lorraine |
| PLT T6B2 May | *Myotis myotis* | Study of maternity colonies | 2019 | Lorraine |
| Pag T19B2 Oct | *Myotis daubentonii* | Study of maternity colonies | 2019 | Lorraine |
| Pag T17B2 Oct | *Myotis daubentonii* | Study of maternity colonies | 2019 | Lorraine |
| Pag T22B2 Jun | *Myotis daubentonii* | Study of maternity colonies | 2019 | Lorraine |
| Pag T10B3 Jun | *Myotis daubentonii* | Study of maternity colonies | 2019 | Lorraine |
| Pag T6B3 Aug | *Myotis daubentonii* | Study of maternity colonies | 2019 | Lorraine |
| Pag T18B2 Jun | *Myotis daubentonii* | Study of maternity colonies | 2019 | Lorraine |
| Swarming2017 CL31F | *Myotis daubentonii* | Swarming | 2017 | Brittany |
| Swarming2018 FT18 163F | *Myotis daubentonii* | Swarming | 2018 | Brittany |
| Pag T15B1 Jun | *Myotis daubentonii* | Study of maternity colonies | 2019 | Lorraine |
| Swarming2017 CL56F | *Myotis daubentonii* | Swarming | 2017 | Brittany |
| Swarming2017 FT248F | *Myotis myotis* | Swarming | 2017 | Brittany |
| Swarming2017 FT201F | *Myotis emarginatus* | Swarming | 2017 | Brittany |
| Swarming2017 FT213F | *Myotis daubentonii* | Swarming | 2017 | Brittany |
| Swarming2018 FT18.164F | *Rhinolophus ferrumequinum* | Swarming | 2018 | Brittany |
| Swarming2017 FT61F | *Myotis daubentonii* | Swarming | 2017 | Brittany |
| Swarming2018 CL34F | *Myotis daubentonii* | Swarming | 2018 | Brittany |
| Pag T2B1 Jun | *Myotis daubentonii* | Study of maternity colonies | 2019 | Lorraine |

**References**

1. Wakeley PR, Johnson N, McElhinney LM, Marston D, Sawyer J, Fooks AR. Development of a Real-Time, TaqMan Reverse Transcription-PCR Assay for Detection and Differentiation of Lyssavirus Genotypes 1, 5, and 6. J Clin Microbiol. 2005;43: 2786–2792. doi:10.1128/JCM.43.6.2786-2792.2005

2. Merien F, Portnoi D, Bourhy P, Charavay F, Berlioz-Arthaud A, Baranton G. A rapid and quantitative method for the detection of Leptospira species in human leptospirosis. FEMS Microbiol Lett. 2005;249: 139–147. doi:10.1016/j.femsle.2005.06.011

3. Toussaint JF, Sailleau C, Breard E, Zientara S, De Clercq K. Bluetongue virus detection by two real-time RT-qPCRs targeting two different genomic segments. J Virol Methods. 2007;140: 115–123. doi:10.1016/j.jviromet.2006.11.007

4. Drexler JF, Corman VM, Wegner T, Tateno AF, Zerbinati RM, Gloza-Rausch F, et al. Amplification of emerging viruses in a bat colony. Emerg Infect Dis. 2011;17: 449–456. doi:10.3201/eid1703.100526

5. Zeng SQ, Halkosalo A, Salminen M, Szakal ED, Puustinen L, Vesikari T. One-step quantitative RT-PCR for the detection of rotavirus in acute gastroenteritis. J Virol Methods. 2008;153: 238–240. doi:10.1016/j.jviromet.2008.08.004

6. Wang J, Wang J, Li R, Liu L, Yuan W. Rapid and sensitive detection of canine distemper virus by real-time reverse transcription recombinase polymerase amplification. BMC Vet Res. 2017;13: 241. doi:10.1186/s12917-017-1180-7

7. Gouilh MA, Puechmaille SJ, Gonzalez J-P, Teeling E, Kittayapong P, Manuguerra J-C. SARS-Coronavirus ancestor’s foot-prints in South-East Asian bat colonies and the refuge theory. Infect Genet Evol J Mol Epidemiol Evol Genet Infect Dis. 2011;11: 1690–1702. doi:10.1016/j.meegid.2011.06.021

8. Woo PCY, Lau SKP, Chu C, Chan K, Tsoi H, Huang Y, et al. Characterization and complete genome sequence of a novel coronavirus, coronavirus HKU1, from patients with pneumonia. J Virol. 2005;79: 884–895. doi:10.1128/JVI.79.2.884-895.2005

9. Mérien F, Amouriaux P, Perolat P, Baranton G, Saint Girons I. Polymerase chain reaction for detection of Leptospira spp. in clinical samples. J Clin Microbiol. 1992;30: 2219–2224. doi:10.1128/JCM.30.9.2219-2224.1992

10. Bourhy P, Collet L, Clément S, Huerre M, Ave P, Giry C, et al. Isolation and Characterization of New Leptospira Genotypes from Patients in Mayotte (Indian Ocean). PLoS Negl Trop Dis. 2010;4: e724. doi:10.1371/journal.pntd.0000724

11. Zhao J, Sun L, Zhao Y, Feng D, Cheng J, Zhang G. Coronavirus Endoribonuclease Ensures Efficient Viral Replication and Prevents Protein Kinase R Activation. J Virol. 2020. doi:10.1128/JVI.02103-20

12. Gomaa MH, Barta JR, Ojkic D, Yoo D. Complete genomic sequence of turkey coronavirus. Virus Res. 2008;135: 237–246. doi:10.1016/j.virusres.2008.03.020

13. Woo PCY, Lau SKP, Lam CSF, Lau CCY, Tsang AKL, Lau JHN, et al. Discovery of seven novel Mammalian and avian coronaviruses in the genus deltacoronavirus supports bat coronaviruses as the gene source of alphacoronavirus and betacoronavirus and avian coronaviruses as the gene source of gammacoronavirus and deltacoronavirus. J Virol. 2012;86: 3995–4008. doi:10.1128/JVI.06540-11

14. Woo PCY, Lau SKP, Lam CSF, Lai KKY, Huang Y, Lee P, et al. Comparative analysis of complete genome sequences of three avian coronaviruses reveals a novel group 3c coronavirus. J Virol. 2009;83: 908–917. doi:10.1128/JVI.01977-08

15. Dong BQ, Liu W, Fan XH, Vijaykrishna D, Tang XC, Gao F, et al. Detection of a novel and highly divergent coronavirus from asian leopard cats and Chinese ferret badgers in Southern China. J Virol. 2007;81: 6920–6926. doi:10.1128/JVI.00299-07

16. Tao Y, Tong S. Complete Genome Sequence of a Severe Acute Respiratory Syndrome-Related Coronavirus from Kenyan Bats. Microbiol Resour Announc. 2019;8. doi:10.1128/MRA.00548-19

17. van Boheemen S, de Graaf M, Lauber C, Bestebroer TM, Raj VS, Zaki AM, et al. Genomic characterization of a newly discovered coronavirus associated with acute respiratory distress syndrome in humans. mBio. 2012;3. doi:10.1128/mBio.00473-12

18. Wu Z, Yang L, Ren X, He G, Zhang J, Yang J, et al. Deciphering the bat virome catalog to better understand the ecological diversity of bat viruses and the bat origin of emerging infectious diseases. ISME J. 2016;10: 609–620. doi:10.1038/ismej.2015.138

19. Obameso JO, Li H, Jia H, Han M, Zhu S, Huang C, et al. The persistent prevalence and evolution of cross-family recombinant coronavirus GCCDC1 among a bat population: a two-year follow-up. Sci China Life Sci. 2017;60: 1357–1363. doi:10.1007/s11427-017-9263-6

20. Wu Z, Lu L, Du J, Yang L, Ren X, Liu B, et al. Comparative analysis of rodent and small mammal viromes to better understand the wildlife origin of emerging infectious diseases. Microbiome. 2018;6: 178. doi:10.1186/s40168-018-0554-9

21. Li B, Si H-R, Zhu Y, Yang X-L, Anderson DE, Shi Z-L, et al. Discovery of Bat Coronaviruses through Surveillance and Probe Capture-Based Next-Generation Sequencing. mSphere. 2020;5. doi:10.1128/mSphere.00807-19

22. Tao Y, Shi M, Chommanard C, Queen K, Zhang J, Markotter W, et al. Surveillance of Bat Coronaviruses in Kenya Identifies Relatives of Human Coronaviruses NL63 and 229E and Their Recombination History. J Virol. 2017;91. doi:10.1128/JVI.01953-16

23. Pan Y, Tian X, Qin P, Wang B, Zhao P, Yang Y-L, et al. Discovery of a novel swine enteric alphacoronavirus (SeACoV) in southern China. Vet Microbiol. 2017;211: 15–21. doi:10.1016/j.vetmic.2017.09.020

24. Lau SKP, Woo PCY, Li KSM, Huang Y, Wang M, Lam CSF, et al. Complete genome sequence of bat coronavirus HKU2 from Chinese horseshoe bats revealed a much smaller spike gene with a different evolutionary lineage from the rest of the genome. Virology. 2007;367: 428–439. doi:10.1016/j.virol.2007.06.009

25. Lin X-D, Wang W, Hao Z-Y, Wang Z-X, Guo W-P, Guan X-Q, et al. Extensive diversity of coronaviruses in bats from China. Virology. 2017;507: 1–10. doi:10.1016/j.virol.2017.03.019

26. Ar Gouilh M, Puechmaille SJ, Diancourt L, Vandenbogaert M, Serra-Cobo J, Lopez Roïg M, et al. SARS-CoV related Betacoronavirus and diverse Alphacoronavirus members found in western old-world. Virology. 2018;517: 88–97. doi:10.1016/j.virol.2018.01.014

27. Lau SKP, Li KSM, Tsang AKL, Shek C-T, Wang M, Choi GKY, et al. Recent transmission of a novel alphacoronavirus, bat coronavirus HKU10, from Leschenault’s rousettes to pomona leaf-nosed bats: first evidence of interspecies transmission of coronavirus between bats of different suborders. J Virol. 2012;86: 11906–11918. doi:10.1128/JVI.01305-12

28. Falcón A, Vázquez-Morón S, Casas I, Aznar C, Ruiz G, Pozo F, et al. Detection of alpha and betacoronaviruses in multiple Iberian bat species. Arch Virol. 2011;156: 1883–1890. doi:10.1007/s00705-011-1057-1

29. Subudhi S, Rapin N, Bollinger TK, Hill JE, Donaldson ME, Davy CM, et al. A persistently infecting coronavirus in hibernating Myotis lucifugus, the North American little brown bat. J Gen Virol. 2017;98: 2297–2309. doi:10.1099/jgv.0.000898

30. Woo PCY, Lau SKP, Li KSM, Poon RWS, Wong BHL, Tsoi H, et al. Molecular diversity of coronaviruses in bats. Virology. 2006;351: 180–187. doi:10.1016/j.virol.2006.02.041

31. Tang XC, Zhang JX, Zhang SY, Wang P, Fan XH, Li LF, et al. Prevalence and genetic diversity of coronaviruses in bats from China. J Virol. 2006;80: 7481–7490. doi:10.1128/JVI.00697-06

32. Han Y, Du J, Su H, Zhang J, Zhu G, Zhang S, et al. Identification of Diverse Bat Alphacoronaviruses and Betacoronaviruses in China Provides New Insights Into the Evolution and Origin of Coronavirus-Related Diseases. Front Microbiol. 2019;10: 1900. doi:10.3389/fmicb.2019.01900

33. Gloza-Rausch F, Ipsen A, Seebens A, Göttsche M, Panning M, Drexler JF, et al. Detection and prevalence patterns of group I coronaviruses in bats, northern Germany. Emerg Infect Dis. 2008;14: 626–631. doi:10.3201/eid1404.071439
